# Influence of ballroom dancing on fatigue, body image, self-efficacy, and endurance of cancer patients and their partners

**DOI:** 10.1007/s12032-021-01459-0

**Published:** 2021-01-28

**Authors:** S. Thieser, J. Dörfler, I. Rudolph, T. Wozniak, T. Schmidt, J. Hübner

**Affiliations:** 1 Klinik für Innere Medizin II, Hämatologie und Internistische Onkologie, Universitätsklinikum Jena, Am Klinikum 1, 07747 Jena, Germany; 2 Waldburg- Zeil Kliniken, Rehabilitationsklinik Bad Salzelmen, Badepark 5, 39218 Schönebeck, Germany; 3 Arbeitsgemeinschaft Prävention und Integrative Onkologie, Deutsche Krebsgesellschaft, 14057 Berlin, Germany; 4 Supportivangebote Sport- und Bewegungstherapie, Universitätsklinikum Schleswig-Holstein, Campus Kiel, Krebszentrum Nord CCC, 24105 Kiel, Germany

**Keywords:** Cancer patients, Physical activity, Ballroom dancing, Body image, Self-efficacy, Fatigue

## Abstract

Physical activity has a high importance for cancer patients. The present study aimed to evaluate the influence of ballroom dancing on fatigue, body image, self-efficacy, and functional exercise capacity for patients with cancer. We collected data among participants of a regular dance training program for cancer patients with a questionnaire including the Body Image Scale (BIS), Brief Fatigue inventory (BFI), Short Scale for Measuring General Self-efficacy Beliefs (ASKU), and the 6-min walking test (6-MWT). 66 participants took part in the study, and among them, 39 participants also engaged in the 6-MWT: dance experience and the weeks of dance training were significantly associated with a higher walking distance in the 6-MWT. Participants with dance experience showed higher self-efficacy scores than participants without. Participants rated the influence of dancing on partnership positively. However, the dance training had no significant effects on fatigue or body image. Ballroom dancing may improve functional exercise capacity, and dance experience may be associated with a high self-efficacy and active lifestyle, which can be beneficial for cancer patients during and after treatment. Further studies are needed to assess the influence of ballroom dancing more deeply on physical activity and fitness and to learn more on impact on the partnership. As ballroom dancing seems to improve physical activity and wellbeing and can promote intimacy between partners, this intervention has the potential to support cancer survivors in various levels.

**Supplementary Information:**

The online version of this article (10.1007/s12032-021-01459-0) contains supplementary material, which is available to authorized users.

## Introduction

Cancer is an important concern with around half a million new cases every year in Germany [1]. Therefore, quality of life (QoL) among cancer patients has been extensively studied in recent years. Nayak and colleagues showed that a low level of QoL was often associated with physical wellbeing affected by fatigue [2].

Fatigue is the most frequent adverse event reported in cancer patients with over 90% [3] and has been shown to be a consequence of active treatment, but it may also persist long term into post-treatment periods [4]. To improve fatigue the National Comprehensive Cancer Network guidelines recommends non-pharmacological interventions such as physical activity (PA) for all stages of cancer.

Another frequent studied influence on QoL of cancer patients is body image, since the psychological wellbeing is often affected by dissatisfaction with their body [2]. Alterations of the body do affect many patients, because changes can appear across different disease and treatment types [5]. These effects on the body are often leading to a negative body image in cancer patients [6]. There are several studies assessing determinants like age, emotional distress, and socioeconomical status that influence body image in cancer patients [6–11]. Furthermore, the influence of PA was assessed and highlighted as a possible approach to improve patients QoL [12, 13]. Despite the benefits, lack of motivation, physical symptoms, fatigue and lack of time are often barriers to PA [3, 14]. Therefore, it is highly important to develop interventions that are feasible and empower the patients to adhere to recommendations of PA. An important concept in the empowerment of patients is the model of self-efficacy [15, 16].

Dance promotes psychological benefits and improves strength and anatomical flexibility [17]. A recent meta-analysis investigated the feasibility and effectiveness of dance interventions and found its significant positive effect on mobility function and endurance performance in healthy older adults [18].

An intervention, which combines a training feasible for most people with biopsychosocial components might be ballroom dancing [19]. There is only a very limited number of pilot studies on ballroom dancing which show its potential to improve QoL of cancer survivors [20, 21]. In 2015, we started a project which offers regular ballroom dance training to patients and their partners. As we have shown, cancer patients prefer a flexible training with regular classes that is adapted to individual needs and fluctuating forces [20, 21]. In contrast, strict curricula with workout and several trainings per week show a reduced adherence and exclude patients with lower or variable fitness. This flexible training with different levels increases wellbeing significantly [22].

Another unique trait of ballroom dancing is the possibility to address partnership and interpersonal/intimacy concerns by promoting intimacy through physical touch, verbal and non-verbal communication, and by fostering enduring commitment [20, 23].

The aim of the study was to assess the influence of a regular ballroom dancing training on fatigue, body image, and self-efficacy as well as on general physical capacity. The ballroom dance training started 2015 in Berlin, as a private initiative by three of the authors (IR, TW and JH) supported by a foundation.

## Patients and methods

### Participants

Participants were recruited from attendees of the dance training. Data were collected from cancer patients as well as their healthy dance partners out of three groups of the regular dance training. Dance partners could be partners, friends, or relatives. We included patients of every age with various types of cancer and disease status, who were either still in or had finished treatment.

Most participants were attending the training for a longer period prior to the study. Furthermore, throughout the study, new participants took part in the training, mostly after they attended a workshop offered by the founders of the training.

Considering their conditions and appointments for treatment, regular participation was not a requirement in either training or study.

### Training

The training was offered once a week, and it was structured in three different training groups based on learning progress and dance experience. Beginners could join the first group without any prior knowledge on dancing, participants with basic knowledge or a fast learning pace could attend the second group, and participants with experience who did need less instructions would attend the advanced group.

Each training was a 90-min course structured in a warmup, two main parts including repetitions, theoretical and practical instructions, a 15-min break in between and individual pauses as needed. Course contents were standard and Latin dances taught by a qualified professional dance instructor.

### Assessment

We collected data from September 2018 until July 2019 with a self-developed questionnaire and the 6-min walking test. Participants were asked to answer the questionnaire right before training in the first, second, fourth, and again after every 7 weeks of training to a total of eight times. The 6-min walking test was done three times, first in the third week of training, again after 20 weeks, and another after 22 weeks of training.

For the questionnaire, we included three established scales:

*Body Image Scale* (*BIS*)*:* a short 10-item scale for assessing body image changes in cancer patients with high reliability (Cronbach's alpha 0.93) and good clinical validity. It was designed to be applicable regardless of cancer type or treatment situation [5]. The original version was translated to a German version and re-translated into English by another trained English-speaking person. Discrepancies were revised and re-translated from German to English by a third scientist, who did not know the original version or the first translation.

Participants were instructed to rate the answers referring to changes in the past week (0: “not at all”; 1: “a little”; 2: “quite a bit”; 3: “very much”). Healthy persons were asked to leave out questions referring to body image changes as result of disease or treatment.

In case of missing scores, we computed the scores from the mean of the items to which the respondents answered.

*German Version of the Brief Fatigue inventory* (*BFI*): the BFI is a brief 9-item scale originally designed for assessing the fatigue level and severity in cancer patients [24]. Evaluation of the German Version of the BFI concluded it to be reliable (Cronbach's alpha 0.92) and valid for cancer and noncancer patients. It showed minor differences in the validation compared to the original version [25]. A mean BFI fatigue score was calculated from the nine BFI items.

The *Short Scale for Measuring General Self-efficacy Beliefs* (*ASKU*) is a short 3-item scale assessing general self-efficacy expectations with a good reliability (McDonalds's omega 0.81–0.86) and validity [26]. A mean ASKU score was calculated from the sum of scores and the answers given.

The *6-min Walk Test (6-MWT)* is an instrument to assess functional exercise capacity, which was originally developed and validated for cardiac and pulmonary patients [27, 28]. After evaluation of the psychometric properties, the 6-MWT was recommended to assess the status of physical functioning and exercise capacity in cancer patients, since reliability and validity seem comparable to healthy elderly and patients with cardiac or pulmonary diseases [29].

We adapted the American Thoracic Society (ATS) Guidelines [27] for the 6-MWT to make the test feasible to the situation and setting of the study. The testing course was a 24 m (8 m × 4 m) square in the undisturbed training room. The length of the square was marked every 1 m. The turnaround points were marked with obstacles.

Each test took place before training and the participants measured and documented their pulse by themselves. Each test was carried out in a group of 4 to 6 participants, who were instructed to walk the outline of the square as closely as possible and covering as much distance as possible by choosing their own moderate pace during the given time. A standardized encouragement was given [30], and the time remaining was called out every minute. Participants were stopped after 6 min and instructed to measure their pulse while their total walking distance (the 6-MWD) in meters was documented.

In addition, sociodemographic parameters, dance experience, tumor status, and treatment situation were obtained by closed questions. We also obtained data on second diseases and added an open question to self-report physical exercises.

Dance experience and physical activity were self-reported items of the participants. Participants should state their dance experience at the beginning of the study and report the month of dance experience, if applicable. Participants, who reported physical exercise aside the ballroom dancing, were sorted in a dichotomous variable as physical active.

There were neither specific requirements for the period of dance experience nor for the type or frequency of exercise.

A scale exploring the influence of the training on the relationship of patients was also incorporated in the questionnaire.

### Statistics

We utilized IBM SPSS Statistics 25 for the data collection and statistical analysis. For the influence of the dance training on partnership quality, we used descriptive statistics.

However, due to a high number of missing data, the lack of independence between cases, and the heterogeneity of the collective, we decided to do separate multilevel models on the dependent variables “Fatigue,” “Body image,” “Self-efficacy,” and “6-MWT” [31]. Within each model, we tested on the influence of the dance training with primary predictor variables: weeks of training (time variable) and dance experience. We also tested on possible secondary influences due to sex, tumor status (patient or healthy partners), physical exercise, and age. Furthermore, in every multilevel analysis, we tested for possible interactions between the predictors and the time variable. If a predictor shows a significant interaction with the time variable, it means that the groups represented by the predictor variable (for example sex) develop differently over time. If the interactions were not significant, we run the model without it.

For the analysis, we combined the overall 45 training weeks to five groups of 9 weeks of training each. Within these groups, we aggregated the individual mean scores of the weeks within a group.

The 6-MWD is known to be affected by factors like differences in cohort, encouragement, corridor length, and frequency of retest, age, height, weight, as well as gender [27, 32]. Due to the deviating terms of our test conditions and protocol, we did not use reference equations from healthy adults [33–35]. The primary question was whether the participants had a significant improvement in their functional exercise capacity after a few weeks of training. Therefore, we used the absolute values of the 6-MWD for our analysis to express change in their general fitness.

We included data of cancer patients and their healthy partners in the analysis. Their allocation was based on the variable tumor status. Tumor status “yes” indicates a cancer patient, while “no” indicates a partner.

## Results

All in all, 66 participants took part in our study. Demographic data are shown in Table 1.

**Table 1:** Demographic data (*n* = 66)

Characteristic	Patients (*n* = 38)		Partners (*n* = 28)	
	No	%	No	%
Age				
< 45 years	4	10.5	0	0
46–55 years	7	18.4	3	10.7
56–65 years	10	26.3	10	35.7
66–75 years	13	34.2	13	46.4
> 75 years	1	2.6	0	0
No data	3	7.9	2	7.1
Gender				
Female	30	78.9	9	32.1
Male	8	21.1	19	67.9
Types of tumor				
Breast	16	42.1		
and Lung	1	2.6		
and Gynecologic	1	2.6		
and Lymphoma	1	2.6		
and Melanoma	1	2.6		
Colorectal	2	5.3		
and Hepatic	2	5.3		
Pancreatic				
and Esophageal	1	2.6		
Kidney	1	2.6		
Lymphoma	1	2.6		
Melanoma	2	5.3		
Gynecologic	2	5.3		
Oropharyngeal	2	5.3		
Thyroid cancer	1	2.6		
Prostate cancer	2	5.3		
Brain tumor	1	2.6		
Lung cancer	1	2.6		
Time since first diagnosis				
> 1 month and < 1 year	3	7.9		
> 1 year and < 10 years	28	73.7		
> 10 years	6	15.8		
No data	1	2.6		
Previous treatment				
Chemotherapy	2	5.3		
Operation	2	5.3		
+ Radiotherapy	3	7.9		
+ Radio, Endocrine therapy	9	23.7		
+ Radio, Endocrine, Others	2	5.3		
+ Chemo	1	2.6		
+ Chemo, Radio	2	5.3		
+ Chemo, Radio, Endocrine	2	5.3		
Cancer recurrence				
+ OP	1	2.6		
+ OP, Chemo	1	2.6		
+ OP, Radio, Endocrine, Others	1	2.6		
+ OP, Chemo, Radio, Endocrine, Others	1	2.6		
+ Radio, Others	1	2.6		
Metastasis				
+ Endocrine therapy	1	2.6		
+ OP	1	2.6		
+ OP, Radio, Endocrine, Others	1	2.6		
+ OP, Chemo	2	5.3		
+ OP, Chemo, Others	1	2.6		
+ OP, Chemo, Radiotherapy	1	2.6		
+ Cancer recurrence, OP, Chemo	1	2.6		
+ Cancer recurrence, OP, Chemo, Others	1	2.6		
+ Cancer recurrence, OP, Chemo, Radio	1	2.6		
Secondary diseases				
Diabetes	3	7.9	0	0
+ Cardiovascular diseases	1	2.6	0	0
Cardiovascular diseases	1	2.6	0	0
+ Others	3	7.9	1	3.6
Other	7	18.4	3	10.7
No secondary diseases	15	39.5	16	57.1
No data	8	21.1	8	28.6

Nearly one third of the participants were between 56 and 65 years old (*n* = 20; 30.3%) and most were between 66 and 75 years old (*n* = 26; 39.4%). There were more female (*n* = 39; 59.1%) than male (*n* = 27; 40.9%) participants. There were 38 (57.6%) cancer patients and 28 (42.4%) healthy dance partners. Breast cancer and its combination with other tumor types were the most common diagnosis (*n* = 20; 52.6%). A majority of 28 patients (73.7%) were diagnosed over a year ago but less than 10 years ago. Information about secondary diseases was given by 15 patients (39.5%) and 4 partners (14.3%).

Out of the 66 participants, 27 answered the questionnaires only and 39 (23 patients and 16 partners) took part in the 6-MWT as well as the questionnaires distributed.

### Results related to the influence of the dance training

#### Partnership quality

The participants were able to evaluate the influence of the dancing on their relationship on a scale from − 4 to + 4, while a score of 0 means no influence on the relationship. Overall, both partners and cancer patients rated the influence of dancing on their partnership as positive. The healthy partners evaluated the influence on their partnership with 2.03 in the first weeks. The score rose to 3.0 in the last weeks of training, showing a positive trend over time. Although tumor patients scored overall lower values, their mean score of 1.84 in the first weeks also slightly rose to a mean of 2.0 in the last weeks (see Figure S1).

#### Fatigue

Cancer patients started with a mean of 3.89 (SD 2) in their fatigue scores. Healthy partners started with a mean of 2.71 (SD 1.83) in their fatigue scores on a scale from 0 to 10.

The dance training did not have any influence on fatigue since weeks of training (*p* = 0.581), and dance experience (*p* = 0.348) did not significantly predict changes in fatigue (see all predictors in Table 2).

**Table 2:** Estimates of fixed effects on Fatigue

Parameter	Estimate	SD	df	*t*-value	*p*-value	95% CI	
						Lower	Upper
Intercept	8.727	1.670	42.532	5.226	**.000**	5.358	12.096
Week 01–09	0	0					
Week 10–18	.110	.322	88.737	.342	.733	− .530	.751
Week 19–27	− .262	.322	84.871	− .812	.419	− .903	.379
Week 28–36	− .460	.417	88.297	− 1.103	.273	− 1.290	.369
Week 37–45	− .436	.474	88.188	− .920	.360	− 1.378	.506
Sex female vs. male	− .231	.548	36.720	− .422	.676	− 1.341	.879
Tumor status no vs. yes	− .897	.506	37.617	− 1.774	.084	− 1.921	.127
Dance experience no vs. yes	− .457	.481	37.438	− .950	.348	− 1.430	.517
Exercise no vs. yes	1.086	.820	48.917	1.326	.191	− .561	2.734
Age	− .075	.024	42.228	− 3.179	**.003**	− .122	− .027

Significant (*p* < .05) are highlighted in bold font

*CI* confidence interval, *df* degree of freedom, *SD* standard error

#### Body image

Cancer patients started with a mean of 0.89 (SD 0.69) in their body image scores. Healthy partners started with a mean of 0.49 (SD 0.69) in their body image scores on a scale from 0 to 3.

The dance training did not have any influence on body image since weeks of training (*p* = 0.156), and dance experience (*p* = 0.639) did not significantly predict changes in body image (see all predictors in Table S1).

#### Self-efficacy

Cancer patients started with a mean of 4.02 (SD 0.95) in their self-efficacy scores. Healthy partners started with a mean of 4.21 (SD 0.63) in their self-efficacy scores on a scale from 1 to 5.

**Fig. 1:**
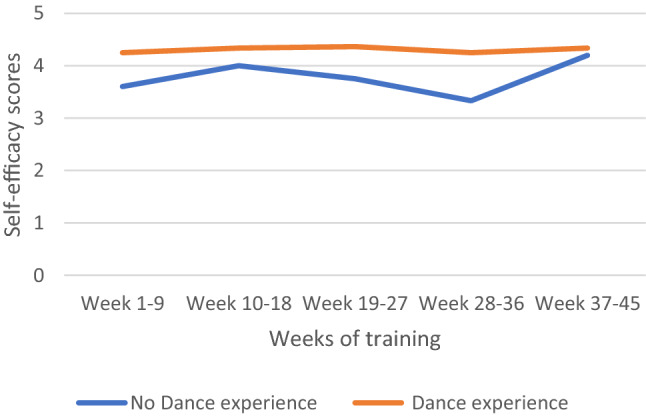
Influence of dance experience on self-efficacy separated for participants with and without former dance experience (*n* = 63). Self-efficacy values could vary between 0 (lowest) and 5 (highest)

There was a significant association between prior dance experience and changes in self-efficacy. On average, participants with no prior dance experience did score 0.64 times lower on self-efficacy than participants with dance experience, [*F* (1, 37.16) = 9.87; *b* = − 0.639, *t* (37.16) =  − 3.14; *p* = 0.003; CI − 1.05, − 0.23] (see Fig. 1 and Table S2).

The weeks of dance training did not influence self-efficacy (*p* = 0.159) (see all predictors in Table 3).

**Table 3:** Estimates of fixed effects on self-efficacy

Parameter	Estimate	SD	df	*t*-value	*p*-value	95% CI	
						Lower	Upper
Intercept	4.538	.709	40.406	6.398	**.000**	3.105	5.971
Week 01–09	0	0					
Week 10–18	.218	.158	86.131	1.384	.170	− .095	.532
Week 19–27	.025	.166	81.801	.148	.883	− .306	.355
Week 28–36	.216	.202	81.895	1.073	.286	− .185	.618
Week 37–45	.070	.214	84.505	.326	.745	− .356	.495
Sex female vs. male	− .251	.237	35.701	− 1.056	.298	− .732	.231
Tumor status no vs. yes	− .060	.243	57.570	− .245	.807	− .547	.427
Dance experience no vs. yes	− .639	.203	37.155	− 3.141	**.003**	− 1.051	− .227
Exercise no vs. yes	.109	.350	45.871	.311	.757	− .595	.812
Age	− .002	.010	40.151	− .245	.807	− .023	.018
Week 01–09 * Tumor status no vs. yes	0	0					
Week 10–18 * Tumor status no vs. yes	− .056	.235	83.178	− .240	.811	− .523	.411
Week 19–27 * Tumor status no vs. yes	.102	.245	81.236	.416	.679	− .385	.588
Week 28–36 * Tumor status no vs. yes	− .821	.304	82.164	− 2.700	**.008**	− 1.425	− .216
Week 37–45 * Tumor status no vs. yes	.194	.358	82.084	.541	.590	− .518	.905

Significant (*p* < .05) are highlighted in bold font

*CI* confidence interval, *df* degree of freedom, *SD* standard error

#### 6-MWT

The 6-MWT was done three times, and all participants were able to complete without resting and no adverse events did occur. Cancer patients started with a mean of 477.1 (SD 50.1) in their walking distance. Healthy partners started with a mean of 471.8 (SD 44.8) in their walking distance.

There was a significant association between the dance training and changes in the walking distance. The more weeks of ballroom dance training the participants did, the more distance they were able to walk in the 6-MWT; [*F* (2, 27.54) = 62.15, *p* = 0.000][(see all predictors in Table 4 and further in Table S3).

**Table 4:** Estimates of fixed effects on 6-MWT

Parameter	Estimate	SD	df	*t*-value	*p*-value	95% CI	
						Lower	Upper
Intercept	658.514	59.614	25.163	11.046	**.000**	535.778	781.251
Week 01–09	0	0					
Week 19–27	2.998	8.730	28.358	.343	.734	− 14.875	20.871
Week 37–45	81.207	9.642	27.317	8.422	**.000**	61.434	100.981
Sex female vs. male	− 44.196	18.405	24.402	− 2.401	**.024**	− 82.149	− 6.242
Tumor status no vs. yes	− 4.631	17.111	25.143	− .271	.789	− 39.863	30.600
Dance experience no vs. yes	− 35.672	18.746	32.294	− 1.903	.066	− 73.842	2.499
Exercise no vs. yes	− 11.241	29.288	23.979	− .384	.704	− 71.692	49.209
Age	− 2.317	.853	25.355	− 2.714	**.012**	− 4.073	− .560
Week 01–09* Dance experience No vs. Yes	0	0					
Week 19–27* Dance experience no vs. yes	59.817	14.588	28.032	4.101	**.000**	29.937	89.697
Week 37–45* Dance experience no vs. yes	22.752	16.525	27.660	1.377	.180	− 11.116	56.620

Significant (*p* < .05) are highlighted in bold front

*CI* confidence interval, *df* degree of freedom, *SD* standard error

**Fig. 2:**
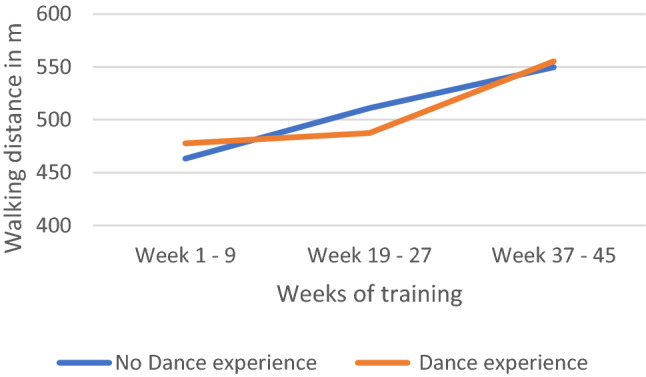
Development of the walking distance (6-MWT) over the training weeks, separated for participants with and without dance experience (*n* = 39)

Furthermore, participants without dance experience and participants with dance experience vary in their walking distance over the course of the training weeks, (*F* (2, 27.69) = 8.51, *p* = 0.001] (see Fig. 2). In the beginning (week 1–9), participants with no dance experience walked almost 35.67 m less than participants with dance experience; [*b* = − 35.672, *t* (32.29) =  − 1.90, *p* = 0.066]. However, with 19 to 27 weeks of training, participants with no dance experience walked 59.82 m more than their counterparts [*b* = 59.817, *t* (28.03) = 4.10, *p* = 0.000, CI 29.94, 89.70]. But then again, in the last weeks, the overall walking distance between the two groups is comparable (*p* = 0.180). Participants with dance experience showed a significant positive development in walking distance from the first weeks to the last weeks of training; [*b* = 81.21, *t* (27.23) = 8.42; *p* = 0.000].

### Results of the not dance-related secondary predictors

#### Fatigue

On average fatigue was significantly lower in older participants than in younger ones. The analysis showed that every additional year of life did predict a 0.075 times lower fatigue value [*F* (1, 42.23) = 10.10; *b* = − 0.075, *t* (42.23) =  − 3.18, *p* = 0.003, CI − 0.12, − 0.03) (see Figure S2).

For all other parameters, we did not find any influence on fatigue: sex (*p* = 0.676), tumor status (*p* = 0.084), and physical exercise (*p* = 0.191) (see all predictors in Table 2).

#### Body image

There were no secondary factors that predicted changes in body image: sex (*p* = 0.148), tumor status (*p* = 0.153), physical exercise (*p* = 0.976), and age (*p* = 0.286) (see all predictors in Table S1).

#### Self-efficacy

Healthy partners and tumor patients did vary in their development of self-efficacy over the course of the training weeks [*F* (4, 80.02) = 2.52; *p* = 0.048] (see Figure S3 and Table S2).

At baseline (week 1–9), the scores of the patients and the healthy partners were comparable, [*b* = − 0.060, *t* (57.57) =  − 0.25; *p* = 0.807, CI = − 0.55, 0.43]. There were no significant differences between the partners and the patients in self-efficacy in week 1 to 27. But after 28 to 36 weeks of training, healthy participants scored 0.82 times lower in contrast to the tumor patients, [*b* = − 0.821, *t* (82.16) =  − 2.70, *p* = 0.008, CI − 1.43, − 0.22].

In the weeks after (37 to 45), there was again no significant difference for self-efficacy values between patients and healthy partners. In conclusion, while tumor patients scored overall slightly higher values over the course of all weeks of training in self-efficacy, the healthy partners did vary in their values, with significant lower values after 28 to 36 weeks of training (see Figure S3).

For all the other parameters, we did not find any significant influences on self-efficacy: sex (*p* = 0.298), physical exercise (*p* = 0.757), and age (*p* = 0.807) (see all predictors in Table 3).

#### 6-MWT

Sex and age did significantly influence the walking distance. Female participants walked overall 44.20 m less than male participants [*F* (1, 24.40) = 5.77, *b* = − 44.196, *t* (24.40) =  − 2.40, *p* = 0.024, CI − 82.15, − 6.24).

Older participants walked less than younger ones. Each year of life did predict a 2.32 m lower walking distances, [*F* (1, 25.36) = 7.37, *b* = − 2.317, *t* (25.36) =  − 2.71, *p* = 0.012, CI − 4.07, − 0.56) (see all predictors in Table 4).

## Discussion

Our study was designed to investigate efficiency of ballroom dancing on cancer patients and their healthy partners. The dance training was introduced as alternative physical activity with positive effects on the patients’ wellbeing and possibly improvement of fitness and partnership quality in our previous studies [21–23].

In this study, we found that prior dance experience and the weeks of dance training were significantly associated with an improvement in functional exercise capacity. Participants with dance experience showed a significant positive development in 6-MWD from the first to the last weeks of the dance training. The participants without dance experience walked less distance in the first weeks of training but showed a significant gain in walking distance after 19 to 27 weeks of training. After 45 weeks of training, all participants showed a higher walking distance, which indicates an improvement in their functional exercise capacity.

This goes in line with the recommendation that exercise should be an essential part of the supportive therapy of cancer patients because of its improvement to physical functioning and QoL and should follow the American College of Sports Medicine (ACSM) guidelines [36, 37]. A systematic review from 2017 showed that exercise at moderate intensity is beneficial in QoL and in muscular and aerobic fitness for patients with cancer-in and post-treatment [38]. Another systematic literature review found that dance of any style improves strength, endurance, and balance as well as functional fitness in elderly [39]. A recent multidisciplinary roundtable concluded that aerobic training as form of endurance training, a combination of aerobic and resistance training, was safe and recommended for cancer survivors [37]. Lankford and colleagues found that the intensity of recreational ballroom dance can be used to meet the physical activity requirements of the ACSM guidelines for improving cardiorespiratory fitness and management of chronic diseases [40]. Ballroom dance training does not only meet the criteria for moderate-vigorous aerobic exercise but seems to be a good choice for long-term compliance of physical activity and participation [40–42].

Studies also showed a superior performance of long-term dancers in comparison to non-dancer control groups on cognitive, motor, and sensory functions, with the implication that advanced dancers maintain an active lifestyle and a general level of physical activity and fitness [19, 43, 44]. The lower walking distance of participants without dance experience in the beginning of our study could be associated with a lower general physical activity. The gain in walking distance after several weeks of training would support the influence of the training on general fitness.

Additionally, we found that dance experience predicted self-efficacy. Since participation with dance experience indicated a certain level of physical activity, this might be explained by a positive correlation between physical activity and self-efficacy found in other studies [45, 46]. Respectively, participants with no dance experience showed lower self-efficacy scores but were still comparable with the means of the general population, whereas the long-lasting dancers in our study had values that were slightly above the means of the general population [26]. This goes in line with the results of various studies that highlight that patients with high levels of self-efficacy effectively engage in exercise, healthy dietary habits and other positive strategic coping behavior [16, 47, 48].

Although studies suggested that physical exercise interventions effectively improve body image among breast cancer patients and cancer survivors [12], neither weeks of dance training, nor other predictors did predict changes in body image in our study. Age could be a contributing factor for this, because studies found that a negative body image and coping with body changes due to cancer treatment were worse in younger woman than in older patients [6, 8, 11, 49]. King and colleagues suggested this may be because of a change in priorities in age and life stage with less importance on physical appearance for their physical attractiveness and femininity [49].

There are different limitations in this study. On the one hand, the trainings course was designed to be flexible and open to the real-life needs of the cancer patients and their partners. On the other hand, this led to a constant fluctuation in participation, with missing data and different starting points in the training. It was, therefore, necessary to modulate the dataset, meaning to sum up the training weeks to have enough participants in each week block for a stable analysis, which led to a loss of individual information per week. The sample was very specific in age, experience of this training, and heterogeneous in terms of cancer type and treatment status. There was no report of the constellation of dance partners, so the healthy partners and cancer patients could not be associated to one another. Therefore, individual changes within the partnership could not be assessed. The 6-MWT had various changes in our protocol in comparison to the official guidelines as reported above. Despite the changes, our study shows similar results to previous papers. Furthermore, the rather long-time interval between the three 6-MWTs was the preferable option for establishing a continuous training over a longer period to improve physical condition since training was offered just once a week.

## Conclusion

The findings of this study support that ballroom dancing may improve functional exercise capacity and long-term participation may be associated with a high self-efficacy and active lifestyle. Further evaluation should investigate the benefits on partnership, fatigue, and body sensation/awareness in cancer patients in a bigger sample with a wider range of age.

## Supplementary Information

Below is the link to the electronic supplementary material.

Electronic supplementary material 1 (DOCX 64 kb)

## References

[1] Barnes B, Kraywinkel K, Nowossadeck E, Schönfeld I, Starker A, Wienecke A (2016). Bericht zum Krebsgeschehen in Deutschland 2016.

[2] Nayak MG, George A, Vidyasagar MS, Mathew S, Nayak S, Nayak BS (2017). Quality of life among cancer patients. Indian J Palliat Care.

[3] Frikkel J, Gotte M, Beckmann M, Kasper S, Hense J, Teufel M (2020). Fatigue, barriers to physical activity and predictors for motivation to exercise in advanced Cancer patients. BMC Palliat Care.

[4] Schmidt ME, Chang-Claude J, Seibold P, Vrieling A, Heinz J, Flesch-Janys D (2015). Determinants of long-term fatigue in breast cancer survivors: results of a prospective patient cohort study. Psychooncology.

[5] Hopwood P, Fletcher I, Lee A, Ghazal SA (2001). A body image scale for use with cancer patients. Eur J Cancer.

[6] Kolodziejczyk A, Pawlowski T (2019). Negative body image in breast cancer patients. Adv Clin Exp Med.

[7] Prates ACL, Freitas-Junior R, Prates MFO, Veloso MF, Barros NM (2017). Influence of body image in women undergoing treatment for breast cancer. Rev Bras Ginecol Obstet.

[8] Paterson CL, Lengacher CA, Donovan KA, Kip KE, Tofthagen CS (2016). Body image in younger breast cancer survivors: a systematic review. Cancer Nurs.

[9] Miller SJ, Schnur JB, Weinberger-Litman SL, Montgomery GH (2014). The relationship between body image, age, and distress in women facing breast cancer surgery. Palliat Support Care.

[10] Chang O, Choi EK, Kim IR, Nam SJ, Lee JE, Lee SK (2014). Association between socioeconomic status and altered appearance distress, body image, and quality of life among breast cancer patients. Asian Pac J Cancer Prev.

[11] Janz NK, Mujahid M, Lantz PM, Fagerlin A, Salem B, Morrow M (2005). Population-based study of the relationship of treatment and sociodemographics on quality of life for early stage breast cancer. Qual Life Res.

[12] Duijts SF, Faber MM, Oldenburg HS, van Beurden M, Aaronson NK (2011). Effectiveness of behavioral techniques and physical exercise on psychosocial functioning and health-related quality of life in breast cancer patients and survivors–a meta-analysis. Psychooncology.

[13] Browall M, Mijwel S, Rundqvist H, Wengstrom Y (2018). Physical activity during and after adjuvant treatment for breast cancer: an integrative review of women's experiences. Integr Cancer Ther.

[14] Pudkasam S, Polman R, Pitcher M, Fisher M, Chinlumprasert N, Stojanovska L (2018). Physical activity and breast cancer survivors: importance of adherence, motivational interviewing and psychological health. Maturitas.

[15] Lee LL, Arthur A, Avis M (2008). Using self-efficacy theory to develop interventions that help older people overcome psychological barriers to physical activity: a discussion paper. Int J Nurs Stud.

[16] Bandura A (1997). Self-efficacy: the exercise of control.

[17] Boing L, Rafael AD, Braga HDO, De Moraes ADJP, Sperandio FF, Guimarães ACDA (2017). Dance as treatment therapy in breast cancer patients—a systematic review. Revista Brasileira de Atividade Física & Saúde.

[18] Liu X, Shen PL, Tsai YS (2020). Dance intervention effects on physical function in healthy older adults: a systematic review and meta-analysis. Aging Clin Exp Res.

[19] Kattenstroth JC, Kalisch T, Holt S, Tegenthoff M, Dinse HR (2013). Six months of dance intervention enhances postural, sensorimotor, and cognitive performance in elderly without affecting cardio-respiratory functions. Front Aging Neurosci.

[20] Pisu M, Demark-Wahnefried W, Kenzik KM, Oster RA, Lin CP, Manne S (2017). A dance intervention for cancer survivors and their partners (RHYTHM). J Cancer Surviv.

[21] Rudolph I, Schmidt T, Wozniak T, Kubin T, Ruetters D, Huebner J (2018). Ballroom dancing as physical activity for patients with cancer: a systematic review and report of a pilot project. J Cancer Res Clin Oncol.

[22] Rudolph I, Dubois C, Schmidt T, Micke O, Wozniak T, Huebner J. Gesellschaftstanz als alternative körperliche Aktivität für Krebspatienten zur Verbesserung ihres Wohlbefindens. Prävention und Rehabilitation. 2020;32(10):251–78. 10.5414/prx0551.

[23] Schmidt T, Rudolph I, Wozniak T, Ruetters D, Van Mackelenbergh MT, Huebner J (2018). Effect of ballroom dancing on the well-being of cancer patients: report of a pilot project. Mol Clin Oncol.

[24] Mendoza TR, Wang XS, Cleeland CS, Morrissey M, Johnson BA, Wendt JK (1999). The rapid assessment of fatigue severity in cancer patients. Cancer.

[25] Radbruch L, Sabatowski R, Elsner F, Everts J, Mendoza T, Cleeland C (2003). Validation of the German version of the brief fatigue inventory. J Pain Symptom Manage.

[26] Beierlein C, Kemper CJ, Kovaleva A, Rammstedt B (2013). Short scale for measuring general self-efficacy beliefs (ASKU). Methods Data Anal.

[27] ATS (American Thoracic Society) (2002). ATS statement: guidelines for the six-minute walk test. Am J Respir Crit Care Med.

[28] Solway S, Brooks D, Lacasse Y, Thomas S (2001). A qualitative systematic overview of the measurement properties of functional walk tests used in the cardiorespiratory domain. Chest.

[29] Schmidt K, Vogt L, Thiel C, Jager E, Banzer W (2013). Validity of the six-minute walk test in cancer patients. Int J Sports Med.

[30] Scheld T (2007). Der 6-Minuten-Gehtest: Ein valides und reliables Verfahren zur Trainingssteuerung und Therapieevaluation in der stationären kardiologischen Rehabilitation. B&G Bewegungstherapie und Gesundheitssport.

[31] Field A (2009). Discovering statistics using SPSS.

[32] Guyatt GH, Pugsley SO, Sullivan MJ, Thompson PJ, Berman L, Jones NL (1984). Effect of encouragement on walking test performance. Thorax.

[33] Enright PL, Sherrill DL (1998). Reference equations for the six-minute walk in healthy adults. Am J Respir Crit Care Med.

[34] Gibbons WJ, Fruchter N, Sloan S, Levy RD (2001). Reference values for a multiple repetition 6-minute walk test in healthy adults older than 20 years. J Cardiopulm Rehabil Prev.

[35] Troosters T, Gosselink R, Decramer M (1999). Six minute walking distance in healthy elderly subjects. Eur Respir J.

[36] Schmitz KH, Courneya KS, Matthews C, Demark-Wahnefried W, GalvÃO DA, Pinto BM (2010). American College of sports medicine roundtable on exercise guidelines for cancer survivors. Med Sci Sports Exerc.

[37] Campbell KL, Winters-Stone KM, Wiskemann J, May AM, Schwartz AL, Courneya KS (2019). Exercise guidelines for cancer survivors: consensus statement from international multidisciplinary roundtable. Med Sci Sports Exerc.

[38] Segal R, Zwaal C, Green E, Tomasone JR, Loblaw A, Petrella T (2017). Exercise for people with cancer: a systematic review. Curr Oncol.

[39] Hwang PW, Braun KL (2015). The effectiveness of dance interventions to improve older adults' health: a systematic literature review. Altern Ther Health Med.

[40] Lankford DE, Bennion TW, King J, Hessing N, Lee L, Heil DP (2014). the energy expenditure of recreational ballroom dance. Int J Exerc Sci.

[41] Young C (2012). The importance of putting the fun back in to youth sports. ACSM's Health Fit J.

[42] Belza B, Walwick J, Shiu-Thornton S, Schwartz S, Taylor M, LoGerfo J (2004). Older adult perspectives on physical activity and exercise: voices from multiple cultures. Prev Chronic Dis.

[43] Kattenstroth JC, Kolankowska I, Kalisch T, Dinse HR (2010). Superior sensory, motor, and cognitive performance in elderly individuals with multi-year dancing activities. Front Aging Neurosci.

[44] Kattenstroth JC, Kalisch T, Kolankowska I, Dinse HR (2011). Balance, sensorimotor, and cognitive performance in long-year expert senior ballroom dancers. J Aging Res.

[45] Awick EA, Phillips SM, Lloyd GR, McAuley E (2017). Physical activity, self-efficacy and self-esteem in breast cancer survivors: a panel model. Psychooncology.

[46] McAuley E, Szabo A, Gothe N, Olson EA (2011). Self-efficacy: implications for physical activity, function, and functional limitations in older adults. Am J Lifestyle Med.

[47] Merluzzi TV, Philip EJ, Heitzmann Ruhf CA, Liu H, Yang M, Conley CC (2018). Self-efficacy for coping with cancer: revision of the Cancer Behavior Inventory (version 3.0). Psychol Assess.

[48] Linde JA, Rothman AJ, Baldwin AS, Jeffery RW (2006). The impact of self-efficacy on behavior change and weight change among overweight participants in a weight loss trial. Health Psychol.

[49] King MT, Kenny P, Shiell A, Hall J, Boyages J (2000). Quality of life three months and one year after first treatment for early stage breast cancer: influence of treatment and patient characteristics. Qual Life Res.

