# Supplementary material for: Influence of ballroom dancing on fatigue, body image, self-efficacy, and endurance of cancer patients and their partners
Source: Med Oncol. 2021 Jan 28;38(2):15. doi: 10.1007/s12032-021-01459-0 (PMC7843482; doi:10.1007/s12032-021-01459-0)
Supplement: Supplementary file 1 — Electronic supplementary material 1 (DOCX 64 kb) [file 12032_2021_1459_MOESM1_ESM.docx]

**Appendix**

**Tabellenverzeichnis**

[Table S 1 Estimates of fixed effects on Body Image 2](#_Toc48736525)

[Table S 2 Interaction: Self-efficacy 2](#_Toc48736526)

[Table S 3 Interaction: 6-MWT 3](#_Toc48736527)

**Abbildungsverzeichnis**

[Figure S 1 Self-reported influence of dancing on partnership quality (n=60) 1](#_Toc48736528)

[Figure S 2 Levels of fatigue in different age groups (n=58) 1](#_Toc48736529)

[Figure S 3 Development of Self-Efficacy during training separated for patients and healthy partners (n=64) 2](#_Toc48736530)

Figure S 1 Self-reported influence of dancing on partnership quality (n=60)

Note: Values could vary from -4 (lowest) to 4 (highest), a score of 0 meant no influence.

Figure S 2 Levels of fatigue in different age groups
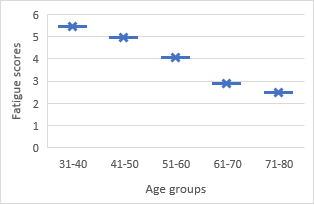
 (n=58)

Note: Fatigue values could vary between 0 (lowest) and 10 (highest)

Figure S 3 Development of Self-Efficacy during training separated for patients and healthy partners (n=64)

Note: Self-Efficacy values could vary between 0 (lowest) and 5 (highest)

| Table S 1 Estimates of fixed effects on Body Image | | | | | | | |
| --- | --- | --- | --- | --- | --- | --- | --- |
| Parameter | Estimate | SD | df | t-value | p-value | 95% CI | |
|  |  |  |  |  |  | Lower | Upper |
| Intercept | 1.356 | .693 | 43.234 | 1.956 | .057 | -.042 | 2.753 |
| Week 01 - 09 | 0 | 0 |  |  |  |  |  |
| Week 10 - 18 | -.100 | .055 | 83.539 | -1.807 | .074 | -.209 | .0100 |
| Week 19 - 27 | -.080 | .056 | 82.854 | -1.420 | .159 | -.192 | .0320 |
| Week 28 - 36 | .062 | .073 | 82.724 | .858 | .394 | -.082 | .207 |
| Week 37 - 45 | -.063 | .080 | 82.951 | -.778 | .439 | -.222 | .097 |
| Sex female vs. male | .351 | .238 | 42.011 | 1.475 | .148 | -.129 | .831 |
| Tumor status No vs. Yes | -.317 | .218 | 42.177 | -1.456 | .153 | -.756 | .122 |
| Dance experience No vs. Yes | .096 | .202 | 42.568 | .473 | .639 | -.312 | .503 |
| Exercise No vs. Yes | -.010 | .334 | 44.575 | -.031 | .976 | -.682 | .662 |
| Age | -.011 | .010 | 43.218 | -1.080 | .286 | -.030 | .009 |

Notes: CI = Confidence Interval, df = degree of freedom, SD = Standard Error. Significant (p < .05) are highlighted in bold front

Table S 2 Interaction: Self-efficacy

| variable | Numerator df | Denominator df | F | p-value |
| --- | --- | --- | --- | --- |
| Relative weeks of training * Sex | 4 | 80.053 | 1.238 | .301 |
| Relative weeks of training * Tumor status | 4 | 80.020 | 2.516 | **.048** |
| Relative weeks of training * Dance experience | 4 | 80.040 | 2.171 | .080 |
| Relative weeks of training * Exercise | 3 | 95.883 | .454 | .715 |
| Relative weeks of training * Age | 4 | 84.559 | .181 | .948 |

*Notes: df = degree of freedom. Significant (p < .05) are highlighted in bold front*

Table S 3 Interaction: 6-MWT

| variable | Numerator df | Denominator df | F | p-value |
| --- | --- | --- | --- | --- |
| Relative weeks of training * Sex | 2 | 30.088 | .152 | .860 |
| Relative weeks of training * Tumor status | 2 | 30.251 | **.034** | .967 |
| Relative weeks of training * Dance experience | 2 | 27.692 | 8.511 | **.001** |
| Relative weeks of training * Exercise | 2 | 29.944 | 1.010 | .376 |
| Relative weeks of training * Age | 2 | 31.780 | 1.931 | .162 |

*Notes: df = degree of freedom. Significant (p < .05) are highlighted in bold front*
